# Molecular characterization of *Salmonella* spp. isolates from river and dam water, irrigated vegetables, livestock, and poultry manures in Jordan

**DOI:** 10.14202/vetworld.2021.813-819

**Published:** 2021-03-31

**Authors:** Yaser H. Tarazi, Abdallah F. Al Dwekat, Zuhair Bani Ismail

**Affiliations:** 1 Department of Veterinary Basic Sciences, Faculty of Veterinary Medicine, Jordan University of Science and Technology, Irbid 22110, Jordan; 2 Department of Veterinary Clinical Sciences, Faculty of Veterinary Medicine, Jordan University of Science and Technology, Irbid 22110, Jordan

**Keywords:** *Salmonella* spp, zoonosis, contaminated river and dam waters, vegetables

## Abstract

**Background and Aim:**

*Salmonellosis* is an important food-borne and zoonotic disease with high morbidity and mortality rates. The objectives of this study were to isolate, serotype, and genetically characterize *Salmonella* spp. from Zarqa river and King Talal dam waters, vegetables irrigated by such waters, and manure of poultry and livestock farms located in the Zarqa river basin in Jordan. In addition, certain virulence factors and antimicrobial resistance patterns of isolated *Salmonella* strains were determined.

**Materials and Methods:**

A total of 250 samples were cultured using routine microbiological methods. Suspected *Salmonella* spp. were identified based on colony morphology and confirmed using biochemical and molecular methods. Virulence genes including *invA, stn, and pCT* plasmid were detected using multiplex PCR. Phylogenetic analysis was performed using pulsed-field gel electrophoresis (PFGE).

**Results:**

In total, 32/250 (12.8%) *Salmonella* spp. isolates were recovered from different sources. Of these, the most common serotype was *Salmonella* subspecies 1 (23 isolates), followed by *Salmonella enterica* serovar Typhimurium (4 isolates), *Salmonella enterica* serovar Typhi (3 isolates), and finally *Salmonella enterica* serovar Enteritidis (2 isolates). The PFGE indicated that *Salmonella enterica* serovar Typhimurium isolated from poultry manure and from parsley were closely related (84.6%). *Salmonella enterica* serovar Enteritidis isolated from the dam water was closely related to *Salmonella enterica* serovar Enteritidis isolated from spearmint (73.8%). *Salmonella enterica* serovar Typhi isolated from the river and dam water were 100% related to *Salmonella enterica* serovar Typhi isolated from lettuce. In the antimicrobial sensitivity test, 14 out of 32 (43.8%) isolated *Salmonella* strains were resistant to two or more of the major antimicrobial agent groups. However, the majority of isolates were sensitive to ceftriaxone, ciprofloxacin, cefuroxime, and gentamicin (97%, 93.8%, and 87.5%, 84.4%, respectively). All isolates were resistant to erythromycin and amoxicillin.

**Conclusion:**

Results of this study indicate a serious potential threat to public health associated with consuming leafy green vegetables grown on the banks of Zarqa river and its dam because of widespread *Salmonella* spp. contamination. Appropriate monitoring of irrigation water must be applied to reduce the possibility of cross-contamination.

## Introduction

Vegetables are one of the most popular raw materials of food because of their convenience and consumer acceptability [1-4]. In the last few decades, the demand for fresh leafy green vegetables has steadily increased due to consumer’s perceived benefits of healthy food for healthier lifestyle [1-4]. Unfortunately, leafy green vegetables are commonly contaminated by various food-borne microorganisms at pre-harvesting, harvesting, or post-harvesting [3,4]. Worldwide, there has been a sharp increase in reported incidences of food-borne illnesses attributed to consumption of raw parsley, lettuce, spearmint, and spinach [3,4]. Sources of contamination are usually soil, irrigation water, organic-based fertilizers, and direct contact with animal feces [1-4].

*Salmonellosis* is considered as one of the most important food-borne and zoonotic diseases causing serious illnesses in humans and animals [5,6]. The primary sources of *Salmonella* organisms in food, including leafy green vegetables, are irrigation using surface water in rivers and dam lakes and the use of organic fertilizers made from poultry or livestock manure [7,8]. The most common food-borne pathogenic *Salmonella* strains have been *Salmonella enterica* serovar *Typhimurium* DT104, *Salmonella enterica* serovar *Enteritidis*, and *Salmonella* subspecies I [9-11].

In Jordan, the Zarqa river and its associated dam (King Talal dam) are major sources of surface water which are mainly used to irrigate vegetables grown alongside its banks and as a drinking water for livestock in the region. The river and dam are notoriously prone to contamination by organic and non-organic material because of their proximity to livestock farms, industrial plants and most importantly, the construction of a major waste water treatment plant where treated wastewater is discharged to the river and ultimately into the dam.The presence of *Salmonella* spp. in the water of the Zarqa river and King Talal dam as well as the prevalence of this important food-borne and zoonotic pathogen in the manure of poultry and livestock and on leafy vegetables in this region has not been studied before. Therefore, the objectives of this study were to isolate, serotyping, and genetically characterize *Salmonella* spp. from Zarqa river and King Talal dam water samples, vegetables irrigated by such waters, and manure of poultry and livestock farms located in the Zarqa river basin. In addition, certain virulence factors and antimicrobial resistance patterns of isolated *Salmonella* strains were determined.

## Materials and Methods

### Ethical approval and Informed consent

No institutional ethical approvals were required in this study since it involved no animal use. However, written and signed consents were obtained from livestock farm owners and vegetable farmers before manure and vegetable samples were collected.

### Study period and location

The study was conducted from June 2015 to May 2016. Laboratory analysis was carried out in the Microbiology Research Laboratory, Faculty of Veterinary Medicine, Jordan University of Science and Technology.

### Sample collection

A total of 250 samples (water, vegetables, poultry, and livestock manure) were collected. The sampled vegetables (n=100; 25 each) were parsley, lettuce, *Eruca sativa*, and spearmint. Water samples (n=50) were directly taken from the river and dam (25 each) from different locations up and downstream. The manure samples (n=100; 25 each) were from poultry, dairy cattle, sheep, and goat farms. All vegetable and manure samples were collected from farms that are located on both sides of the river and used water for irrigation or drinking for animals from the river or from the dam. Fresh green leaves and vegetable samples were collected using sterile forceps and scissors and put into sterile polyethylene bags. For each manure sample, 100 g of manure were collected from 5 points in each farm, one sample from the center, and four from periphery of the farm by sterile spatula, mixed, and placed in a sterile fecal cup container [12].

### Bacterial isolation and identification

To culture vegetable samples, a 25 g of fresh sample was homogenized with 225 mL of buffered peptone water (BPW; Oxoid) in a stomacher (Seward Stomacher, England) and incubated at 37°C for 18 h. Then, 2 aliquots (0.1 mL) were inoculated in Rappaport Vassiliadis soy peptone (RVS) broth (Oxoid) and Tetrathionate broth (TTB; Oxoid) and incubated at 41.5°C and 37°C, respectively, for 24 h. Then, 10 mL aliquots from RVS and TTB broths were inoculated on Xylose Lysine Deoxycholate (XLD; Oxoid) and on Brilliant Green agar (BGA; Oxoid) and incubated at 37°C for 24 h.

For water samples, 1 ml of each water sample was inoculated into 10 ml BPW (Oxoid) and incubated at 37°C for 18 h. Then, two aliquots (0.1 mL) were inoculated in RVS broth (Oxoid) and TTB broth (Oxoid) and incubated at 41.5°C and 37°C, respectively, for 24 h. Then, 10 mL aliquots from RVS and TTB broths were inoculated on XLD (Oxoid) and on BGA (Oxoid) and incubated at 37°C for 24 h.

For manure samples, 1 g of each sample was inoculated into 10 ml BPW (Oxoid) and incubated at 37°C for 18 h. Then, two aliquots (0.1 mL) were inoculated in RVS broth (Oxoid) and TTB broth (Oxoid) and incubated at 41.5°C and 37°C, respectively, for 24 h. Then, 10 mL aliquots from RVS and TTB broths were inoculated on XLD (Oxoid) and on BGA (Oxoid) and incubated at 37°C for 24 h.

Presumptive identification of *Salmonella* spp. was made initially based on colony morphology and Gram staining characteristics. Confirmation was then achieved by biochemical methods (RapID ONE System, Thermo Fisher, USA) according to manufacturer’s instructions. Pure colonies of the isolates were then transferred into slants of nutrient agar (Oxoid), incubated at 37°C for 24 h and stored as stock cultures until further use [12].

For control, the reference strains obtained from the American Type Culture (ATCC) 14028 (*Salmonella enterica* serovar Typhimurium), ATCC 13076 (*Salmonella enterica* serovar Enteritidis) and National Collection of Type Cultures (NCTC) 5760 (*Salmonella enterica* serovar Typhi), and NCTC 8385 (*Salmonella* subspecies I) were used in the study.

### DNA extraction

DNA was extracted by the boiled cell method [13]. Briefly, 1 mL of each isolate broth was centrifuged for 3 min at 15,000 g. The supernatant was then discarded and the pellet was resuspended in 500 mL of sterile distilled water and vortexed. The cell suspension was boiled for 10 min and immediately cooled at −20°C for 10 min, followed by centrifugation at 15,000 g for 3 min. The supernatant was transferred into clean tubes to be used as the DNA template solution for the optimization of the multiplex PCR.

### Detection of virulence factors

PCR was performed to detect *Salmonella*-specific *invA* gene (responsible for cell invasion), enterotoxin gene (*stn*), and the *pCT* plasmid gene using commercially available primers (Table-1). The PCR reaction solution was made of the following components: 0.5 mL of each primer, 12.5 mL Dream Taq master mix (Promega, USA), and 3 µL template DNA, nuclease-free water were then added to reach total volume of 25 mL. For negative control, sterile distilled water instead of the template DNA solution was included in three wells in each PCR assay. The PCR machine (Thermal Cycler; Applied Biosystems, CA) program was set at 5 min at 94°C before 35 cycles of 1 min at 94°C, annealing temperature at 55°C for *stn* and *invA*, extension at 72°C for 1 min, and a final extension of 10 min at 72°C. For *pCT* plasmid gene, the same conditions were used except the annealing temperature was 60°C for 1 min [7,14]. All PCR products were analyzed by gel electrophoresis in 2% agarose gels and stained with 0.5 mg/mL ethidium bromide, and then visualized under ultraviolet (UV) light [15]. A 100-bp ladder was used as a marker in all electrophoresis gel runs.

**Table-1:** Sequences of primers of various virulence genes used in the study to characterize *Salmonella* isolates from River and Dam water samples, irrigated vegetables, and poultry and livestock manure samples in Jordan [7,14].

Gene	Sequence	Size (bp)
*stn*	F- TTGTGTCGCTATCACTGGCAACC R- ATTCGTAACCCGCTCTCGTCC	617
*invA*	F- GTGAAATTATCGCCACGTTCGGGCAA R- TCATCGCACCGTCAAAGGAACC	284
*pCT*	F- CATTGTATCTATCTTGTGGG R- GCATTCCAGAAGATGACGTT	428

### Serotyping of *Salmonella* isolates

Serotyping of *Salmonella* isolates was performed using multiplex PCR optimized for the detection of *Salmonella enterica* serovar Typhimurium, *Salmonella enterica* serovar Enteritidis, *Salmonella enterica* serovar Typhi, and *Salmonella* subspecies I using the primer pairs as shown in Table-2. The primer pairs were evaluated in the final multiplex PCR reaction using three repetitions to ensure reproducibility of the assay. The PCR reaction was made of a 25 mL containing 0.5 mL of each primer pair, 12.5 mL Dream Taq master mix (Promega, USA), 3 mL templates DNA, and nuclease-free water to reach the total volume 25 mL. For negative control, sterile distilled water instead of the template DNA solution was included in three wells in each PCR assay. The PCR machine (Thermal Cycler; Applied Bio systems, CA) program was set at 94°C 3 min, annealing temperature 57°C 30 s and 72°C 30 s, for 30 cycles, and final extension 72°C 3 min and was stored at 4°C until used. All PCR products were analyzed by gel electrophoresis in 2% agarose gels and stained with (0.5 mg/mL) ethidium bromide, and then visualized under UV light [15]. A 100-bp ladder was used as a marker in all electrophoresis gel runs.

**Table-2:** Primers used in the serotyping of *Salmonella* isolates by multiplex PCR [15].

Serotype	Sequence	Size (bp)
*Salmonella enterica* serovar Typhi	F- TTACCCCACAGGAAGCACGC R- CTCGTTCTCTGCCGTGTGGG	203
*Salmonella enterica* serovar *Typhimurium*	F- AACAACGGCTCCGGTAATGAGATTG R- ATGACAAACTCTTGATTCTGAAGATCG	310
*Salmonella enterica* serovar *Enteritidis*	F- GGATAAGGGATCGATAATTGCTCAC R- GGACTTCCAGTTATAGTAGGTGGCC	559
*Salmonella* subspecies I	F- GGTGGCCTCGATGATTCCCG R- CCCACTTGTAGCGAGCGCCG	137

### Antimicrobial sensitivity test

The antimicrobial sensitivity test was performed using the agar disk diffusion method on Mueller-Hinton agar plates (Oxoid) [16]. The inoculated plates were incubated at 35°C for 24 h. A total of 14 antibiotics were tested using commercially available disks (Thermo Fisher) including ciprofloxacin (5 mg), cefuroxime (30 mg), sulfamethoxazole/trimethoprim (23.75/1.25 mg), tetracycline (30 mg), nalidixic acid (30 mg), chloramphenicol (30 mg), ceftriaxone (30 mg), kanamycin (30 mg), penicillin (10 IU), gentamicin (10 mg), oxytetracycline (30 mg), streptomycin (10 mg), erythromycin (15 mg), and amoxicillin (20 mg). Results of the sensitivity test were classified as sensitive, intermediate, or resistant according to the diameter of the inhibition zone according to the National Committee of Clinical Laboratory Standards guidelines [16].

### Pulsed field gel electrophoresis (PFGE)

#### Bacterial culture preparation

All isolates were subcultured on tryptone soya *agar* plates (Oxoid) and incubated at 37°C for 18 h to obtain a confluent growth. All colonies were removed from the surface of TSA plates using sterile polyester fiber applicator moistened with sterile cell suspension buffer (CSB; 100 mM Tris: 100 mM Ethylenediaminetetraacetic acid [EDTA], pH 8.0) and transferred into sterile tubes containing 2 mL of CSB. The concentration of each cell suspension was adjusted to a value correspondent to an absorbance value of 1.3-1.4 O.D. at 610 nm wavelength using a spectrophotometer (Jenway, UK). A 400 mL aliquot of each adjusted cell suspension was then transferred to a sterile microcentrifuge tube containing 20 mL of proteinase K (20 mg/mL stock) then the 400 mL of melted 1% SeaKem Gold agarose (Sigma, USA) prepared in TE buffer (10 mM Tris:1 mM EDTA, pH 8.0) was added and the mixture was mixed by gentle pipetting up and down (melted agarose was kept at 5560°C in water bath). After that, the mixture was dispensed into the wells of PFGE plug and the plugs were allowed to solidify at 4°C for 5 min. The plugs were then removed from the molds and placed in a 5 mL tube containing 5 mL of Cell Lysis Buffer (CLB; 50 mM Tris, 50 mM EDTA, pH 8.0), 1% Sarcosyl (Sigma), and proteinase K (0.1 mg/mL proteinase K) and incubated at 54°C in shaker water bath for 2 h. After that, the plugs were washed 2 times with sterile ultrapure water pre heated to 50°C for 15 min in a water bath and followed by four washes with sterile TE buffer (TE; 10 mM Tris: 1 mM EDTA, pH 8.0) preheated to 50°C for 15 min. The plugs were stored in TE buffer at 4°C until used.

### Restriction digestion with XbaI enzyme

Two-millimeter-wide piece was cut from each of the plugs and added to a tube containing 200 mL of a 1× dilution of the restriction buffer and incubated at 37°C water bath for 5-10 min. The restriction buffer was replaced with 200 mL of *XbaI* restriction enzyme and incubated for 2 h at 37°C; then the restriction mixture was replaced with 200 mL of 0.5× Tris borate EDTA (TBE; prepared from 10-TBE containing 0.89 M Tris borate, 0.02 M EDTA, pH 8.3) and allowed to stand for 5 min to saturate the plug slices with electrophoresis running buffer.

### Electrophoresis conditions and casting of the agarose gel

The 1% SKG gels (Sigma) plugs were loaded in the electrophoreses wells and the electrophoresis conditions for *Salmonella* spp. were set as follows: Initial switch time of 2.2 sec and a final switch time of 63.8 s (based on a fragment range of 30-700 kb) and electrophoresis run time of 18-19 h. After the electrophoresis was completed, the gels were stained with 400 mL of ethidium bromide solution (dilute 40 mL of ethidium bromide stock solution [10 mg/mL] with 400 mL of distilled water for 20-30 min), then the gel was de-stained in 500 mL distilled water for 60-90 min, and then the image was captured using video gel documentation system (Bio-Rad, USA). The image was then analyzed using the Jaccard band coefficient to calculate the similarities between the lanes, with a tolerance of 0.625% and the relatedness between isolates was determined using BioNumerics version 2 Software (Applied Maths, Belgium).

## Results

Out of 250 samples, 32 (12.8%) isolates were positively confirmed as *Salmonella* spp. using biochemical and molecular methods. Of these, 16 isolates (50%) were from the river, dam, spearmint, and lettuce (4 each); three isolates (9.38%) were from *E. sativa*, two isolates (6.25%) from parsley, seven isolates (21.87%) from poultry manure, three isolates (9.38%) from goat manure, one isolate (3.12%) from sheep manure, and no isolates were cultured from dairy cow manure. According to the source of the samples, *Salmonella* spp. were recovered in 16% (8/50), 11% (11/100), and 13% (13/100) from the river and dam water combined, poultry, and livestock manure combined and vegetables combined, respectively. All isolates were positive for the *invA* and *stn* genes, except 1 isolate from goat manure which was negative for *stn* gene. None of the isolates carried the *pCT* plasmid gene.

**Figure-1:**
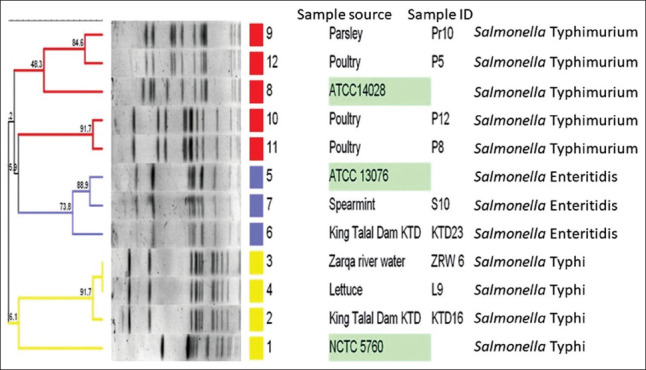
Phylogenic analysis of *Salmonella* spp. isolated from Zarqa River water, dam water, vegetables, and poultry manure showing two main clusters with close similarities between isolates from different sources.

Serotyping indicated that the most common serotype was *Salmonella* subspecies I (23 isolates), followed by *Salmonella enterica* serovar Typhimurium (four isolates), *Salmonella enterica* serovar Typhi (three isolates), and finally *Salmonella enterica* serovar Enteritidis (two isolates). Table-3 shows the distribution of *Salmonella* serotypes according to the sample source in the studied area. The PFGE analysis revealed that isolated strains were of two major clusters with all *Salmonella enterica* serovar Typhi located in one cluster while all *Salmonella enterica* serovar Typhimurium and *Salmonella enterica* serovar Enteritidis isolates were located in the second cluster (Figure-1). The PFGE indicated that *Salmonella enterica* serovar Typhimurium isolates from poultry manure and from parsley were closely related (84.6%). *Salmonella enterica* serovar Enteritidis isolated from the dam was closely related to *Salmonella enterica* serovar Enteritidis isolated from spearmint (73.8%). *Salmonella enterica* serovar Typhi isolates from lettuce and from the river water were 100% related to *Salmonella enterica* serovar Typhi isolated from the dam water.

**Table-3:** Serotyping of *Salmonella* spp. isolated from Zarqa river water, King Talal dam water, vegetables, poultry, and livestock manure using multiplex PCR technique.

Sample sources	Serotypes (n = 32)			

	*Salmonella enterica* serovar Typhi (n = 3)	*Salmonella enterica* serovar Typhimurium (n = 4)	*Salmonella enterica* serovar Enteritidis (n = 2)	*Salmonella* subspecies I (n = 23)
Zarqa River water	1	0	0	2
King Talal dam water	1	0	1	4
Vegetables	1	1	1	11
Poultry manure	0	3	0	2
Livestock manure	0	0	0	4

In the antimicrobial sensitivity test, 14 out of 32 (43.8%) isolated *Salmonella* strains were resistant to three or more of the antimicrobial groups (multidrug-resistant) (Table-4). The majority of isolates were sensitive to ceftriaxone, ciprofloxacin, cefuroxime, and gentamicin (97%, 93.8%, 87.5%, and 84.4%, respectively). All isolates were resistant to erythromycin and amoxicillin.

**Table-4:** Antimicrobial sensitivity patterns of *Salmonella* spp. isolated from Zarqa river basin in Jordan (n=32).

Antimicrobial agents	Sensitivity patterns (%)		

	Sensitive	Intermediate	Resistant
Ciprofloxacin (5 mg)	30 (93.8)	2 (6.3)	0
Cefuroxime (3 0 mg)	28 (87.5)	3 (9.4)	1 (3)
Sulfamethoxazole/trimethoprim (23.75/1.25 mg)	20 (62)	0	12 (37.5)
Tetracycline (30 mg)	14 (43.8)	2 (6.3)	16 (50)
Nalidixic acid (30 mg)	21 (65.6)	2 (6.3)	9 (28)
Chloramphenicol (30 mg)	18 (56)	0	14 (43.8)
Ceftriaxone (30 mg)	31 (97)	0	1 (3)
Kanamycin (30 mg)	22 (68.8)	7 (21.9)	9 (28)
Streptomycin (10 mg)	20 (62)	2 (6.3)	10 (31.3)
Penicillin (10 IU)	4 (12.5)	7 (21.9)	21 (65.6)
Gentamicin (10 mg)	27 (84.4)	0	5 (15.6)
Oxytetracycline (30 mg)	7 (21.9)	4 (12.5)	21 (65.6)
Erythromycin (15 mg)	0	0	32 (100)
Amoxicillin (20 mg)	0	0	32 (100)

## Discussion

This is the first study to investigate the presence of *Salmonella* pathogens in two main surface water sources in Jordan used for irrigation of crops and leafy green vegetables and in adjacent livestock farms. Furthermore, the study determined the prevalence of different *Salmonella* serotypes, virulence characteristics, antimicrobial sensitivity patterns as well as their genetic relatedness and to determine the degree of cross-contamination. Overall, 12.8% (32/250) of all samples contained at least one serotype of pathogenic *Salmonella* spp. including *Salmonella enterica* serovar Typhimurium, *Salmonella enterica* serovar Typhi, and *Salmonella enterica* serovar Enteritidis or *Salmonella* subspecies I. In the current study, the rate of *Salmonella* spp. recovered from the water of Zarqa river and King Talal dam was 16% (8/50), which is higher than the incidence rate (11%) of *Salmonella* contamination in surface water found in farms in New York State [17]. Surface water sources used for irrigation consistently appear to be a major reservoir for *Salmonella* spp. all over the world to a certain degree, the rate of which may be influenced by rainfall runoff and drainage from livestock farms, and wastewater management plants [18]. In this study, the relatively high rate of *Salmonella* spp. recovery from Zarqa River and King Talal dam may be attributed to dumping of treated wastewater from the nearby wastewater treatment plant, water runoffs from untreated or imperfectly treated wastes from towns and rural areas, and livestock farm wastes [19].

The rate of *Salmonella* spp. recovered in this study from poultry manure (2.8%) was lower than that reported elsewhere in the world [20]. In a small ruminant research farm in Virginia State University, *Salmonella* spp. was detected in 3.5% of fecal samples which is higher than our finding [21]. It has been stated that the runoff water and waste from livestock farms to rivers and lakes and the use of contaminated animal manure as an organic fertilizer may results in cross-contamination of crops and vegetables with *Salmonella* spp. [1-4]. Moreover, it has been found that *Salmonella* can persist in the farm environment for extended periods of time due to circulation within the farm between different pools such as animals, livestock excrement, soil, and plants [22]. In this study, 13% of leafy green vegetables were contaminated with *Salmonella*. These results are similar to previously reported data in Nigeria (13.9%) [12]. The much lower isolation rate of *Salmonella* spp. (1.8%) has been reported from leafy green vegetables in Catalonia, Spain [23]. On the other hand, in Egypt, the presence of *Salmonella* was found in 42% in lettuce, 29% in strawberries [24]. The contamination of leafy green vegetables planted in Zarqa river basin and Jordan valley is most likely due to irrigation from contaminated surface water of Zarqa river and King Talal dam. Furthermore, presence of untreated animal manure in Zarqa river basin may have contaminated the river water that ultimately results in leafy green vegetables contamination. Hence, improper manure handling can be an important cause of direct contamination of water supplies and indirectly contaminate irrigated vegetables.

In the current study, *Salmonella enterica* serovar Typhimurium, *Salmonella enterica* serovar Typhi, *Salmonella enterica* serovar Enteritidis, and *Salmonella* subspecies I were detected in manure, water, and leafy green vegetables and most of them were detected in summer (May to September). All *Salmonella* isolates were resistant to one or more major groups of antibiotics. However, none of the isolates carried the multidrug-resistant plasmid gene (*pCT*). This is may be due to other intrinsic resistance factors. These results are closely similar to previously reported data where most *Salmonella* isolates recovered from surface water, manure, and leafy green vegetables were resistant to multiple antibiotics [7]. It has been reported that some bacteria spp. may develop resistance to multiple antibiotics by spontaneous mutations that may occur within certain chromosomes [25]. This type of mutation may then transmit vertically and horizontally under the effect of antibiotic selective pressure [25].

In this study, there was considerable relatedness between different *Salmonella* strains isolated from different sources. This may indicate the survival and persistence of this bacterium in contaminated water sources and animal manure with a very likely potential for cross-contamination to leafy green vegetables. These results are in complete agreement with those reported previously, where high relatedness was found between *Salmonella* isolates obtained from vegetables, water, animals, and human [26]. Similar results were also reported among *Salmonella* isolates obtained from food, environmental and clinical samples [27].

## Conclusion

Results of this study showed that two of the major water sources in Jordan that are currently used to irrigate crops and used in livestock farms are contaminated with pathogenic strains of *Salmonella* spp. Genetic analysis in this study indicated that the use of animal manure as a fertilizer of upstream lands, rain, and waste water runoffs as potential sources of this bacteria. Therefore, the use of such low-quality surface water for irrigation of crops is considered a potential source of *Salmonella* cross-contamination to human food supply, including leafy green vegetables. This presents a real and serious threat to human health because of the high pathogenicity and multiple drug resistance characteristics of the isolated *Salmonella* strains. It is recommended to monitor the quality of wastewater, runoff water, and surface water sources used for irrigation of crops and vegetables in this region to prevent cross-contamination of human food with this pathogenic bacterial.

## Authors’ Contributions

YHT: study concept and design. AFA: sample collection and performed laboratory testing. ZBI: scientific advice, data interpretation, and manuscript writing. All authors read and approved the final manuscript.
